# Microbiota and Other Preventive Strategies and Non-genetic Risk Factors in Parkinson’s Disease

**DOI:** 10.3389/fnagi.2020.00012

**Published:** 2020-03-12

**Authors:** Rafael Franco, Rafael Rivas-Santisteban, Irene Reyes-Resina, Gemma Navarro, Eva Martínez-Pinilla

**Affiliations:** ^1^Chemistry School, University of Barcelona, Barcelona, Spain; ^2^Centro de Investigación Biomédica en Red Enfermedades Neurodegenerativas (CiberNed), Instituto de Salud Carlos III, Madrid, Spain; ^3^Department of Biochemistry and Physiology, Faculty of Pharmacy and Food Science, University of Barcelona, Barcelona, Spain; ^4^Departamento de Morfología y Biología Celular, Facultad de Medicina, Universidad de Oviedo, Oviedo, Spain; ^5^Instituto de Neurociencias del Principado de Asturias (INEUROPA), Oviedo, Spain; ^6^Instituto de Investigación Sanitaria del Principado de Asturias (ISPA), Oviedo, Spain

**Keywords:** cognitive reserve, gut microbiota, dysbiosis, pesticides, methylxanthines, substantia nigra, dopaminergic neurons

## Abstract

The exact cause of Parkinson’s disease (PD), the second most prevalent neurodegenerative disease in modern societies, is still unknown. Many scientists point out that PD is caused by a complex interaction between different factors. Although the main risk factor is age, there are other influences, genetic and environmental, that individually or in combination may trigger neurodegenerative changes leading to PD. Nowadays, research remains focused on better understanding which environmental factors are related to the risk of developing PD and why. In line with the knowledge on evidence on exposures that prevent/delay PD onset or that impact on disease progression, the aims of this review were: (i) to comment on the non-genetic risk factors that mainly affect idiopathic PD; and (ii) to comment on seemingly reliable preventive interventions. We discuss both environmental factors that may affect the central nervous system (CNS) or the intestinal tract, and the likely mechanisms underlying noxious or protective actions. Knowledge on risk, protective factors, and mechanisms may help to envisage why nigral dopaminergic neurons are so vulnerable in PD and, eventually, to design new strategies for PD prevention and/or anti-PD therapy. This article reviews the variety of the known and suspected environmental factors, such as lifestyle, gut microbiota or pesticide exposition, and distinguishes between those that are harmful or beneficial for the PD acquisition or progression. In fact, the review covers one of the most novel players in the whole picture, and we address the role of microbiota on keeping a healthy CNS and/or on preventing the “side-effects” related to aging.

## Introduction

Parkinson’s disease (PD) is one of the most prevalent diseases in advanced societies whose main risk factor is age. Two types can be distinguished based on the onset age. Early-onset PD (EOPD), i.e., clinical symptoms occurring before 50 years of age (even before 40), is mainly due to genetic alterations. However, the percentage of patients carrying mutations in PD-related genes is much lower than that of the idiopathic PD cases, whose clinical symptoms usually start past the age of 60.

PD is a well-defined disease that consists of the death of dopamine-producing neurons in the substantia nigra (SN). Like other synucleinopathies, it is characterized by aggregations of α-synuclein in the so-called Lewy bodies. The resulting lack of dopamine in motor control areas of the brain leads to the characteristic trends: tremor, difficulty to start any automatized movement, etc. In the long run, patients develop cognitive alterations that may be quite severe. The still pending question is why these neurons die or, in other words, why are these neurons so vulnerable to genetic alterations or environmental factors? The impact of PD-related genetic factors (Polymeropoulos, 1998; Valente et al., 2004; Eriksen et al., 2005; Deng et al., 2006; Spatola and Wider, 2014; Franco et al., 2019) on these neurons will be covered in other articles in the Research Topic and, therefore, is out of the scope of this review article.

Fortunately, PD research has led to efficacious therapeutic interventions. The first one, which is still in use, is the therapy with levodopa that, after its intake, is converted into dopamine (Birkmayer and Hornykiewicz, 1962, 1964; Hornykiewicz, 2006); this is known as the “substitutive therapy.” Similar, though less used or used only when levodopa therapy is not working properly, is the treatment with dopamine receptor agonists. In any case, medication has to be chronic, and this leads to undesirable side-effects, the most common being levodopa-induced involuntary movements (dyskinesias; Olanow et al., 2004). When such side-effects severely affect the daily activities there is still an efficacious therapy, already available in the nineties, consisting of brain surgery to implant electrodes in the subthalamic nucleus whose electric activity is controlled by pacemaker-like devices placed under the skin. Using the right parameters, the intervention, known as deep brain stimulation (DBS), produces a re-balancing of motor-control circuits to afford dyskinesia disappearance and normal deambulation (Benabid et al., 1994; Duff and Sime, 1997; Obeso et al., 1997; Tasker et al., 1997; Kumar et al., 1998; Walter and Vitek, 2004; Guridi et al., 2018). Any good news, such as therapies that are not available for other neurodegenerative diseases like Alzheimer’s, is obscured by the lack of any intervention that is able to stop disease progression. Unfortunately, there is no interventions to stop or delay the progressive death of neurons for PD, Alzheimer’s, Huntington’s, or other neurodegenerative diseases. At this point, potentially useful strategies include avoiding risk factors and looking for actions that can be preventive.

The aim of this review article is double-sided. On the one hand, we will cover some of the known or suspected environmental factors that may reduce the survival of nigral dopaminergic neurons. On the other hand, we will comment on the opposite i.e., which lifestyle and surrounding factors can be preventive. Finally, we think that it is of interest to comment on a new player in this conundrum, namely microbiota. Gut microbiota is becoming popular as, depending on its nature, it may provide benefits or alterations at the whole-body level (including brain).

## Influence of Pesticides and Related Environmental Toxics in Parkinson’s Disease Onset

There are surely gene–environment links in PD pathophysiology (Singh et al., 2014; Karimi-Moghadam et al., 2018), i.e., two individuals with different genomes exposed to the same pesticide may respond differently, and, in the same way, two different pesticides may differentially affect a given genome. As an example, allelic variants of GSTP1, coding for glutathione S-transferase P, and CYP2D6, coding for cytochrome P_450_, impact on the differential sensitivity to pesticides (Singh et al., 2014). In summary, individuals with the “inappropriate” combination of alleles in these genes are more sensitive to environmental factors, and pesticides are among the best characterized in PD physiopathogenesis.

To date, the most commonly used PD animal models rely on providing a neurotoxin that kills dopaminergic neurons. These models have been instrumental to the advancement of knowledge of PD pathophysiology. Toxicity may be achieved by proximity delivery, such as through an intracerebroventricular injection of 6-hydroxy-dopamine (Ungerstedt, 1971; Carey, 1986a, b; Andén et al., 1966) or, systemically, by oral or intraperitoneal administration of a toxic compound. Interestingly for the aim of this article, toxins used may provide further information on risk assessment. The use of 1-methyl-4-phenyl-1,2,3,6-tetrahydropyridine (MPTP) to generate rodent and non-human primate PD models (Langston et al., 1984; Johannessen et al., 1985; Jenner and Marsden, 1986; Snyder and D’Amato, 1986; Williams, 1986; Tipton and Singer, 1993; Schulz et al., 1995; Przedborski et al., 1996; Ara et al., 1998) gives a clue: affected cells must have the DAT dopamine transporter, i.e., unlike neurons unable to uptake MPTP, dopaminergic neurons are the most affected. Taking into consideration all data concerning MPTP and rotenone (see below) effects, it is clear that toxicity requires their incorporation into cells, likely altering the energy metabolism of dopaminergic neurons.

Rotenone is another of the toxics that already in 1963 was known to be an inhibitor of the main provider of energy of cells in aerobiosis: the mitochondrial electron transport chain (Chance et al., 1963). In 1985, Emmanuel Geoffrey discovered a compound in *Lonchocarpus nicou*, Nicouline (see Ambrose and Haag, 1936). Later, Nagai Nagayoshi isolated “rotenone” from *Derris elliptica* (see Laforge et al., 1933). Although the two compounds are identical, the name given by the Japanese chemist prevailed. Rotenone was used for centuries as a non-selective pesticide, and it was not until 2007 that it was labeled an environmental toxin by the Environmental Protection Agency of the United States of America. Compound exposure is considered a risk factor for PD, something that fits with PD-like symptoms when the compound is administered to a mammal and with the long-time exposure of humans to this, now forbidden, pesticide. In fact, one of the existing PD animal models consists of rodents exposed to rotenone (Heikkila et al., 1985).

Lindane, an organochlorine pesticide (γ-hexachlorocyclohexane), was associated with convulsions due, most likely, to either heavy doses or repeated exposure (Gupta, 1975). Despite the relatively few reports on lindane and PD, there is solid evidence pointing to a link between exposure to certain types of insecticides and PD risk. A relevant piece of information came from determining the content of lindane in the SN of post-mortem samples from PD, dementia with Lewy bodies, Alzheimer’s disease, and age-matched non-PD (non-demented) controls. The level of lindane was significantly higher in the SN of PD patients (Corrigan et al., 2000). In a similar study but using the now forbidden environmental hazard dieldrin [(1R, 2S, 3S, 6R, 7R, 8S, 9S, 11R)-3, 4, 5, 6, 13, 13-hexachloro-10-oxapentacyclo(6.3.1.13, 6.02, 7.09, 11)tridec-4-ene], authors found the compound in the brain of 6 out of 20 PD cases but in none of the control brains (Fleming et al., 1994). Despite some controversy due to the difficulty in performing accurate epidemiological studies (Costa, 2015), there is solid evidence linking PD risk to the exposure of similar chemicals used for pest control. In a case-control study, serum β-hexachlorocyclohexane levels were associate with PD diagnosis (Richardson et al., 2011). Laboratory data from blood and hair collected for various individuals proved that the odds ratio for PD was statistically significant for β-hexachlorocyclohexane (Petersen et al., 2008). In the case of seizures, it was suggested in the early seventies that the mechanism of toxicity was dependent on the ammonia and glutamine accumulation with the order of toxicity: lindane > dieldrin > heptachlor (1, 5, 7, 8, 9, 10, 10-heptachlorotricyclo[5.2.1.02, 6]deca-3, 8-diene) > DDT (1,1′-(2,2,2-Trichloro-1,1-ethanediyl)bis(4-chlorobenzene; Omer, 1971). However, the mechanism in PD seems different; the fact that MPTP is toxic only after entering the cell and rotenone is toxic for mitochondrial function suggests that mitochondria-related events play a significant role. Thus, exposure to certain heavy metals increases PD risk likely by interfering with Fe^2+/^Fe^3+^ conversions in hemoglobin, in mitochondrial cytochromes or both. Potential risk factors are, among others, manganese, copper, and bismuth (Levin and Sukhotina, 1956; Gibbs and Walshe, 1971; Smyth et al., 1973; Bahiga et al., 1978; Torkian et al., 2019). In the latter, PD was developed after parenteral treatment of alveolar pyorrhoea with bismuth salts (Galata, 1964). Independently of environmental factors, excess absorption of iron from food, coming from to blood transfusions, thalassemia, etc., causes hemosiderosis, which gives rise to several serious consequences and one of them may be parkinsonism (Asenjo et al., 1968; Aracena et al., 2006; Jiang et al., 2019; Thirupathi and Chang, 2019). Details on the links between metal-based PD risks and/or underlying mechanisms may be found elsewhere (Cannon and Greenamyre, 2012; Bjorklund et al., 2018; Ball et al., 2019; Thirupathi and Chang, 2019).

Pioneering studies in late eighties discovered a deficient activity of nicotinamide adenine dinucleotide (NADH)-ubiquinone (or coenzyme Q) reductase, i.e., of complex I of mitochondrial electron chain transport, in platelets of 10 PD patients (Parker et al., 1989). Actually, the deficit in complex I activity was soon discovered in the SN from post-mortem samples of nine PD patients (comparing with samples from age-matched controls; Schapira et al., 1990). Authors concluded that: “*These results indicated a specific defect of Complex I activity in the SN of patients with PD. This biochemical defect is the same as that produced in animal models of parkinsonism by MPTP and adds further support to the proposition that PD may be due to an environmental toxin with action(s) similar to those of MPTP*.” Further studies confirmed mitochondrial abnormalities in the periphery using platelet-rich preparations from another cohort of patients (Krige et al., 1992; Schapira et al., 1992) or blood lymphocytes (Barroso et al., 1993). Authors concluded that: “*our study supports the hypothesis that a biochemical defect in the respiratory chain may be involved in the pathogenesis of PD*.” Although it may not happen in all PD cases, mitochondrial alterations are found often, and they are transversal, i.e., they may be found in both early onset and ideopathic PD cases, in transgenic models, and in non-transgenic animal models (Mizuno et al., 1995; Hauser and Hastings, 2013; Karimi-Moghadam et al., 2018).

Genetic polymorphisms in mitochondrial DNA have been associated with PD. As an example (Chu et al., 2015) reported that an A10398G polymorphism and two haplotypes associated with either 10398G or 10398A are risk factors for the development of sporadic PD in a relatively homogenous population in Northern China. Interestingly, this association was stronger in females than in males. Transcriptomics analysis at the single cell level using post-mortem SN samples from patients have also detected alterations in the amount of gene products encoded in mitochondrial DNA (Grünewald et al., 2016). Moreover, in Pink1^−/−^ transgenic PD models, which lack the E3 ubiquitin ligase parkin, there is a decrease in the level of free complex I while the energy metabolism is compromised (Lopez-Fabuel et al., 2017). Again, the evidence points towards mitochondrial components and events as direct players in almost any PD-associated risk (Vanitallie, 2008). One of the transgenic models is based on this knowledge; the “MitoPark” mouse model, developed by inactivation of mitochondrial transcription factor A, a protein essential for mitochondrial function, displays a progressive PD-like phenotype (Ekstrand and Galter, 2009).

Another important consequence of mitochondrial malfunctioning is oxidative stress. While one may consider whether oxidative stress is a consequence of dopaminergic neuron death (Sanders and Greenamyren, 2013), all the evidence points to complex I defective function leading to oxidative stress. Many environmental risk factors affect the mitochondria (Abdulwahid Arif and Ahmad Khan, 2010). Unfortunately, the possibility of coenzyme Q supplementation (Beal, 1999; Ebadi et al., 2001) and the promising results of a phase II trial (Shults, 2005) did not translate into benefits for patients (Negida et al., 2016), thus suggesting that coenzyme Q is not the limiting factor able to rescue mitochondrial malfunctioning in neurons of the SN.

In summary, the most reasonable hypothesis is that age and exposure to environmental factors, with or without particular polymorphisms, lead to reduced electron transport, reduced ATP production and increased oxidative stress. These events are detrimental for every cell in the human body, but it seems that nigral neurons are more vulnerable. The Ockham’s Razor rule indicates that these neurons lack appropriate detoxification mechanisms, and/or that the demanding metabolism related to dopamine production makes rescue mechanisms lose their efficacy upon aging.

## Dysbiosis as Risk a Factor of Parkinson’s Disease

Dysbiosis of gut microbiota refers to the pathological imbalance between the beneficial flora, i.e., Lactobacteria species, Bifidobacterium, or Enterococci, and opportunistic bacteria, i.e., Bacteriodes, Clostridia, Enterobacteria, Staphylococci, or Streptococci, living permanently or occasionally in the human gut (Roy Sarkar and Banerjee, 2019). This aberrant condition leads to gastrointestinal, metabolic and various neurodegenerative disorders, PD among them. It should be noted that dopamine, which was thought to be mainly produced in the central nervous system (CNS), is found at relatively high levels in the gastrointestinal tract (Eaker et al., 1988).

Regarding neurodegenerative diseases, different studies have evidenced an intimate bidirectional communication between gut microbiota and CNS. On one hand, the so-called Gut-Brain Axis links the sympathetic and parasympathetic nervous system of the gut with the CNS. On the other hand, CNS regulates gut function (immunity, permeability, or mucus secretion) *via* afferent and efferent autonomic pathways (Collins and Bercik, 2009; Carabotti et al., 2015).

Gut dysbiosis and the consequent disruption of beneficial relationship between gut microbiota and CNS may impact on PD progression. In fact, some authors suggest that the neurodegenerative cascade in PD could start in the gastrointestinal tract as a result of an increased gut permeability, inflammation and oxidative stress. This may explain the intestinal alterations such as recurrent constipations observed in patients at very early stages of PD, long before the characteristic motor symptoms (Houser and Tansey, 2017; Parashar and Udayabanu, 2017; Nair et al., 2018).

Pioneering studies comparing gut flora composition of PD patients with age-matched healthy controls found higher levels of *Enterobacteriaceae* species and lower abundance of *Prevotellaceae* (Scheperjans et al., 2015). *Prevotellaceace* family members contribute to keep digestive structures in optimal conditions by the production of mucin and short chain fatty acids (SCFAs; Nair et al., 2018). It is proved that decreases in *Prevotellaceae* species alter gut permeability and lead to systemic exposure to bacterial endotoxins (Roy Sarkar and Banerjee, 2019). Moreover, other bacteria species with probed anti-inflammatory properties, such as *Blautia*, *Coprococcus* and *Roseburia*, are significantly diminished in PD patients (Felice et al., 2016; Parashar and Udayabanu, 2017). *Enterobacteriaceae* population seems to be related with inflammation processes, postural instability, and motor dysfunction in PD (Mukherjee et al., 2016; Houser and Tansey, 2017). Guo et al. (2013) demonstrated that lipopolysaccharide (LPS) derived from the cell wall of gram-negative bacteria compromises intestinal epithelial barrier and makes a possible entrance into the blood stream of LPS and potential neurotoxics. Disruptions to the gut permeability and systemic exposure to bacterial antigens induce the expression of inflammatory cytokines, such as tumor necrosis factor (TNF-α) or interleukin (IL)-1β and IL-6, that alter the blood–brain barrier (BBB), promote α-synuclein accumulation in the SN and lead to dopaminergic cell death (Singh et al., 2014). Other determining factors in dopaminergic neuron loss may be the small intestinal bacterial overgrowth, termed SIBO, displayed by PD patients. Satisfactory treatment of SIBO is related to the long-lasting improvement in motor and intestinal symptoms of patients, and this is probably due to greater efficiency in levodopa absorption (Anderson et al., 2016; Felice et al., 2016; Parashar and Udayabanu, 2017).

According to the hypothesis of Braak et al. (2006), the pathological accumulation of α-synuclein could start in the enteric nervous system (SNE) spreading to the brain through the vagus nerve (Braak et al., 2006). In this sense, histologically identical inclusions to Lewy bodies have been found in enteric neurons of both submucosal (Meissner) and myenteric (Auerbach) plexuses following a hypothetical rostral-caudal gradient; a higher concentration of α-synuclein in the submandibular gland and esophagus and a lower concentration in the colon and rectum were found (Felice et al., 2016). However, other studies suggest that intestinal disturbance is a consequence of CNS damage (Mulak and Bonaz, 2015).

Along PD progression gut microbiota could also contribute to the synthesis of key metabolites and neuroactive compounds. An example of pivotal relevance is the case of tryptophan catabolites (TRYCAT). In the gastrointestinal tract tryptophan can be metabolized using three different routes: (i) direct catabolism into indole and tryptamine; (ii) the kynurenine pathway; and (iii) the serotonin pathway. In the kynurenine route, L-tryptophan is metabolized into kynurenine in two sequential steps catalyzed by indoleamine 2,3-dioxygenase (IDO) and tryptophan 2,3-dioxygenase (TDO). Kynurenine is further metabolized into several metabolites that play an important role in immunoregulation, neurotransmission and inflammation. Other metabolites derived from microbial action on tryptophan can act as potent neurotoxins (Anderson et al., 2016; Agus et al., 2018).

Recent studies have shown that patients with PD exhibit higher proportion of L-kynurenine/tryptophan in serum. This finding may result from enhanced IDO and TDO enzymatic activities in patients. In addition, increased levels of 3-hydroxykynurenine, a neurotoxic TRYCAT, have been found in the putamen, prefrontal cortex, and SN of patients with PD (Calabrese et al., 2018). It is important to note that the intestinal production of melatonin through the serotonin pathway may be downregulated due to increased IDO and TDO activities (Agus et al., 2018). This neuromodulator has some important physiological functions, such as being anti-inflammatory and also acting as an antioxidant, and it has even been reported to counteract the neurotoxicity of α-synuclein deposition. Lower levels of melatonin and its receptors are common in the SN of PD brains.

Finally, and although PD-related genetic factors are out of the scope of this review, we do not want to overlook recent studies suggesting that interactions between host genetics and microbial exposures could contribute to some chronic diseases such as inflammatory bowel disease (IBD) and PD. In this sense, it has been demonstrated that some microbiota alterations produce PD-like symptoms in genetically susceptible individuals (Knights et al., 2014; Palm et al., 2014; Matheoud et al., 2019).

Bearing in mind all the data, it seems that maintaining a balanced gut microbiota could minimize the pathological processes of PD and even be a preventive factor. In this sense, consumption of probiotics containing *Lactobacillus casei shirota* significantly improves gastrointestinal symptoms in patients with PD (Felice et al., 2016). One interesting study showed that regular intake of *Bacillus spp*. may increase dopamine levels since this probiotic bacterium can convert L-tyrosine into levodopa (Parashar and Udayabanu, 2017). A recent innovative treatment linking microbiota and PD is the fecal microbiota transplantation by which feces from healthy donors are transferred to the gastrointestinal tract of a PD patient in order to restore their microbiota. It has been described that healthy mice carrying feces from patients with PD show motor alterations and a reduction of *Lachnospiraceae* and *Ruminococceae* populations, similar to those observed in PD patients (Parashar and Udayabanu, 2017). However, fecal microbiota transplants remain unapproved by US Food and Drug Administration (FDA), and further studies are necessary to probe their benefits as a therapy to combat/prevent PD (Tremlett et al., 2017).

## Inflammation and Infection as Risk Factors of Parkinson’s Disease

Depending on the pathogen, infections affect different mammalian body components. Most infectious agents, even viruses or bacteria that do not lead to neurological symptoms may reach the brain. Fortunately, in the majority of cases the brain does not suffer gross anatomical alterations and/or significant disturbances in a higher function. However, as many pathogens cause inflammation and PD courses with neuroinflammation, not only in animal models of the disease but also in patients, the hypothesis of a link between infection and PD risk was emitted. It is very difficult to assess in humans whether a given viral infection increases the PD risk. Also, it is difficult to perform a study in patients trying to look for the occurrence of “similar” infections. Every human suffers in her/his lifetime a myriad of infections and the number of pathogens to which a human is exposed is counted by dozens. In summary, it may be that a given pathogen increases PD risk but to identify it is like finding a needle in a haystack. To our knowledge, there is no intervention aimed at avoiding infections that could result in an increased risk of suffering the disease, i.e., of leading specifically to the death of SN dopaminergic neurons. However, it should be noted that an infection caused by the hepatitis C virus seems to be a risk factor for PD (Benito-León, 2017; Wijarnpreecha et al., 2018). But even in the case that a relationship between a viral infection and PD risk can be found, it is difficult to address the mechanisms by which viral-induced immunoreaction would affect more dopaminergic cells of the SN than other neuronal types. At present, it is assumed that inflammation, represented by activated glial cells, specially by reactive microglia, is a consequence of dopaminergic cell death. Facts and hypotheses encompassing the noxious role attributed to activated glial cells, prompted the approach of using anti-inflammatory drugs to delay disease progression. The rationale was that reducing inflammation would result in a decline in the rate of neuronal death.

Despite promising trends in animal models, all the efforts to try anti-inflammatory drugs to produce signs of improvement to PD patients have failed. Details may be found in the data deposited in the US clinical trial database^1^. In terms of risk assessment, one of the first meta-analyses that included data from seven studies concluded that: “*There may be a protective effect of nonaspirin nonsteroidal anti-inflammatory drug use on risk of PD consistent with a possible neuroinflammatory pathway in PD pathogenesis*” (Gagne and Power, 2010). This cautionary conclusion has not been validated in further studies, such as the one by Ren et al. (2018) who, upon considering 15 studies, concluded that the consumption of non-steroidal anti-inflammatory drugs is not associated with PD risk (Ren et al., 2018). The most recent work including 17 studies with 14,713 patients within *circa* 2,5 million participants also failed to find an association between non-steroidal anti-inflammatory drugs and PD risk at the population level (Poly et al., 2019). Authors riveted: “*clinicians need to be vigilant ensuring that the use of NSAIDs remains restricted to their approved anti-inflammatory and analgesic effect*.”

## Emerging Concepts of Lifestyle and Cognitive Reserve in Parkinson’s Disease Development

Current research about aging and aging-associated neurodegenerative diseases focuses on the lifestyle factors and on cognitive reserve, a concept that has been proposed to explain the discrepancies between the degree of brain damage and the observable cognitive manifestations. Overall, cognitive reserve encompasses two different but complementary components—cognitive reserve and brain reserve—that have been used to account for inter-individual differences in cognition deficits. On the one hand, *brain reserve* refers to some neurobiological aspects such as brain volume, number of neurons, or number of synapsis or dendritic branches that impact on the “brain resilience” to age-related changes. Instead, *cognitive reserve* refers to the plastic changes occurring from all experiences along a life: formal education, emotional support, level of occupation, work position, familial relationships, physical activity or leisure activities. It is hypothesized that promoting mental activity in the course of our lives can influence our structural neuronal network complexity and our functional processing capacity and efficiency, which in turn can activate compensatory mechanisms that prevent injury (see Barulli and Stern, 2013; Sampedro-Piquero and Begega, 2017). Extensive experimental evidence, starting with the “nuns” study and going all the way to the Honolulu-Asia Aging Study (Latimer et al., 2017), has confirmed that a higher cognitive reserve is associated with less cognitive decline and a delay in the appearance of clinical symptoms in neurodegenerative diseases (Stern, 2002, 2009; Valenzuela and Sachdev, 2006). Also, the comparative analysis of the press conferences of two US presidents, in order to afford an early diagnosis of a degenerative disease, is of interest (Alzheimer’s in the study by Berisha et al., 2015).

The effects of cognitive reserve in PD have raised the interest of many researchers in the last decade, as well as some controversy. A significant number of studies report that lower education levels, measured as the number of years in education, are associated with an increased risk of dementia in PD (Poletti et al., 2011). Inversely, cognitive reserve may slow the progression of global cognitive decline in PD showing a positive effect (Cohen et al., 2007; Poletti et al., 2011; Koerts et al., 2013; Hindle et al., 2015). It is not clear whether there is enough empirical evidence behind these findings. Thus, one cross-sectional study of 120 PD patients reported that higher education level may exert protective effect only in short-term memory and not on the global cognitive decline; while a systematic meta-analysis did not find an association between cognitive reserve and lower risk of long-term dementia in PD (Pai and Chan, 2001; Hindle et al., 2014). Noteworthy, some studies, as the one carried out by Muslimović et al. (2009), show that years of daily brain stimulation positively correlated with cognitive performance of PD patients. Therefore, it seems that “poor” neuronal stimulation can be associated with higher cognitive decline (reasoning, attention, processing speed, or memory; Muslimović et al., 2009).

Although the benefits of intellectual stimulation on cognitive function have been clearly demonstrated in environmental enrichment (EE) interventions both in animals and in some clinical trials in humans, a number of outstanding questions about how cognitive reserve modulates cognitive decline in PD have yet to be answered. What is the most appropriate age to start brain stimulation? Or what is the minimum level of education, i.e., years of formal education, that translates into an improvement of cognitive function? A better understanding of cellular and molecular mechanisms underlying cognitive stimulation may provide valuable information about how cognitive reserve modulates cognitive decline and counteracts neurodegenerative processes in PD-like conditions.

Behavioural benefits of EE and how these interventions correlate with plastic changes at the circuit and cellular levels can be assessed in animal models. In this sense, it is shown that the positive effects of EE modify the structure of the brain, and EE increases, among others, the volume and weight of the hippocampus and of the cortical and some subcortical areas. In addition, EE may be also able to trigger the expression of neurotrophic factors of great relevance for the maintenance of neuronal homeostasis, neurogenesis, neuronal survival and synaptic structure and function. Important factors are the brain-derived neurotrophic (BDNF), the nerve growth (NFG), the glial cell-derived neurotrophic (GDNF) and the vascular endothelial growth (VEGF) factors. The overexpression of some genes related with formation and consolidation of new synapses must be taken into account (Sampedro-Piquero and Begega, 2017). Interestingly, in a recent work, authors suggested a potential implication of microglia in the beneficial effect of long-term EE in diseases coursing with neuroinflammation and in systemic metabolism (Ali et al., 2019). In contrast, serious alterations in neuronal activity and even cell death have been observed in the case of animals exposed to impoverished environments (Muslimović et al., 2009; Sampedro-Piquero and Begega, 2017).

## Preventive Interventions for Parkinson’s Disease

Neuroprotective interventions are difficult to identify and are restricted to a few conditions. There are, for instance, the so-called nootropics that eventually prevent dementia. In the case of PD, epidemiological interventions that had improved over time have led to identifying some substances that reduce the risk of suffering from the disease.

### Caffeine and Other Methylxanthines

Some historical background will be helpful to understand why caffeine and related methylxanthines (theophylline and theobromine) are PD preventive. Methylxanthines are non-selective antagonists of adenosine receptors and it turns out that one of them, the A_2A_ receptor (A_2A_R) is highly enriched in striatum (Rosin et al., 1998), i.e., in the main target structure of any pharmacological anti-PD treatment. The role of endogenous adenosine in the basal ganglia is to counteract the effect of dopamine. In particular, activation of the A_2A_R by adenosine reduces dopamine D_2_-receptor-mediated transmission (Fuxe et al., 1983; Zoli et al., 1993; Hillion et al., 2002). Accordingly, blockade of the A_2A_R receptor by an antagonist increases dopaminergic effects. A long time ago, it was hypothesized that adenosine receptor antagonists would be beneficial to combat PD, if combined with dopaminergic agonists or with levodopa. After many years of research and of clinical trials with up to five different novel A_2A_R antagonists, one of them was approved (Nouriast^TM^) in Japan as adjuvant to levodopa in PD therapy (Jenner et al., 2009; Mizuno and Kondo, 2013; Saki et al., 2013; Kondo and Mizuno, 2015). The same compound has been recently approved by the FDA and prescribed as Nourianz^TM^. Importantly, research on A_2A_R and PD has led to the discovery that genetic ablation of the receptor or its pharmacological blockage is neuroprotective in animal models. At present, there is no suitable protocol to assess neuroprotection in humans (even in patients with neurodegenerative diseases). Alternatively, one may assume that “chronic” consumption of A_2A_R antagonists could be neuroprotective. Actually, coffee/tea are considered parameters to assess the risk of developing PD in relation with dietary habits. The report by Ragonese et al. (2003) constitutes a good example of early studies with solid evidence that drinking coffee prevents PD.

There is controversy in almost any potential risk factor. For instance, despite previous works providing evidence on negative correlation between coffee consumption and PD risk (Sipetic et al., 2012), we have been unable to find similar results in a case-control study. On the one hand, there is a consensus that there are compounds in coffee that can afford protection, such as caffeine and similar methylxanthines, which block adenosine receptors. On the other hand, the consumption of caffeine is not easy to asses due to differences in caffeine content in coffee, to inter-country differences in the style/amount of drinking coffee/tea, and to the fact that caffeinated drinks must be also considered in the risk analysis. Overall, the evidence stands up. In the exhaustive meta-analysis of Noyce et al. (2012), a significant negative association between coffee drinking and PD risk is confirmed. More information on the validity of this negative association can be found in more recent reviews (Ascherio and Schwarzschild, 2016; Chen, 2019).

Although cacao/chocolate and tea have caffeine and other methylxanthines and there are cultural differences in the amount of consumed coffee, tea, caffeinated beverages, etc., Eskelinen et al. (2009, 2011) proved, within the Cardiovascular Risk Factors, Aging and Dementia (CAIDE) study, that mid-life sustained consumption of caffeine and tea is protective in dementia and Alzheimer’s disease (Eskelinen and Kivipelto, 2010; Sindi et al., 2018). One of the hypothesis behind the benefits of sustained methylxanthine intake for the CNS, also backed up by the fact that virtually all societies have methylxanthine-containing beverages, is that active neurons are less prone to death. Adenosine receptor antagonism produces neuronal activation and, eventually, active neurons have more tools to cope with environmental factors leading to oxidative stress (Franco et al., 2013; Oñatibia-Astibia et al., 2017).

### Smoking

In past times, it was usual to smoke while taking coffee. This fact has prompted the search for the relationship between smoking and PD risk. The first results were positive but they were challenged by the idea that smokers live less and, therefore, they are less susceptible to suffer from an age-related disease. A case-control study with males of the same socio-cultural context showed that PD patients had more exposure to smoking (>10 cigarettes/day) and drinking (>50 g ethanol/day) habits. Taken together, the results in the literature are interesting. Some of the early studies found that 63% of cases (*n* = 237) and 47% of controls (*n* = 474) never smoked (Baumann et al., 1980). In the *Drosophila* *melanogaster* model of PD, nicotine-free tobacco affords neuroprotection. The translatability of this result to patients is dubious, as the same study reports that caffeine-free coffee is neuroprotective (Trinh et al., 2010). To our knowledge, the more informative article is constituted by a systematic review of the literature and a meta-analysis. Authors found significant differences: the reported risk reduction was 36% for individuals with a smoking habit. They also indicated that “*the effect is strongest in current smokers and weakest in past smokers (56% for current smokers and 22% for past smokers), but the association remains significant in all*” (Noyce et al., 2012). In the same year, a case control study also found that smoking was PD protective (Sipetic et al., 2012). In summary, at present there are little doubts that smoking cigarettes protects against suffering from PD and there are hints in animal models that support the most obvious hypothesis, i.e., that nicotine in tobacco is the relevant factor for PD risk reduction (for review, see Ma et al., 2017).

The mechanism of protection of nicotine and caffeine (or other methylxanthines) may be different. On the one hand, nicotine affects nicotinic ionotropic receptors and there are no reports showing nicotine effects *via* direct action on adenosine receptors. On the other hand, nicotine produces addiction and, hence, the neuroplasticity in the rewarding areas that could impact on PD risks does not occur upon consumption of coffee, cola drinks, etc.

### Urate

The Honolulu Heart Program, which consisted of a 30-year prospective epidemiological study, incidentally discovered an association between serum levels of urate, above the median, and less PD risk (Davis et al., 1996). Afterwards, the seminal clinically-oriented work of Schwarzschild et al. (2008) proved that the concentration of urate in blood plasma/serum is a parameter to consider in PD. More importantly, increased values of urate seem to be preventive although it is known that excess production of uric acid leads to deposition in joints and to a clinical condition known as gout. First of all, we would like to highlight that urate is a reliable marker of disease progression (Schwarzschild et al., 2008, 2011; Ascherio et al., 2009; Cipriani et al., 2010; Crotty et al., 2017; Paganoni and Schwarzschild, 2017). In addition, relatively high urate levels in men’s serum are associated with less PD risk (Weisskopf et al., 2007; Gao et al., 2008; Bronstein et al., 2009; Cipriani et al., 2010; Ascherio and Schwarzschild, 2016; Paganoni and Schwarzschild, 2017; Kim et al., 2018). The reasons are not totally clear but it may be related to oxidative stress, that is specially detrimental in highly active cells such as those producing dopamine in the SN (Crotty et al., 2017). Also, another intriguing point is the poor association existing in females as reported by the same research team (O’Reilly et al., 2010). Interestingly, inosine administration to elevate urate levels in early PD female patients (intervention within the SURE-PD trial) showed a slower clinical decline (Schwarzschild et al., 2019).

## Author Contributions

Conceptualization was agreed by RF, GN and EM-P, who also scanned the literature, retrieved and extracted information from articles referenced in the review. RR-S and IR-R analyzed the literature and provided stratification reports on PD risk/prevention. RF compiled all the information and wrote the first version of the manuscript. GN and EM-P critically read the manuscript and prepared a second version of the article. All authors edited the manuscript and revised the final submitted version.

## Conflict of Interest

The authors declare that the research was conducted in the absence of any commercial or financial relationships that could be construed as a potential conflict of interest.

## Footnotes

^1^https://clinicaltrials.gov/
